# 异基因非亲缘非HLA匹配脐带血输注治疗难治性免疫性血细胞减少症：Ⅰ期临床试验结果

**DOI:** 10.3760/cma.j.issn.0253-2727.2023.05.014

**Published:** 2023-05

**Authors:** 青林 胡, 冰 韩, 伟汉 何, 辰 杨, 苗 陈

**Affiliations:** 中国医学科学院北京协和医学院北京协和医院血液科，北京 100730 Department of Hematology, Peking Union Medical College Hospital, Peking Union Medical College, Chinese Academy of Medical Sciences, Beijing 100730, China

免疫性血细胞减少症（immune mediated cytopenia, IMC）是一类自身免疫介导的血细胞减少疾病，包括自身免疫性溶血性贫血（AIHA）、Evans综合征、免疫性血小板减少症、纯红细胞再生障碍（PRCA）、结缔组织病相关免疫性血细胞减少等，主要采用糖皮质激素及免疫抑制剂治疗[1]–[2]。对于糖皮质激素耐药、依赖（泼尼松维持剂量>15 mg/d）的患者，长期免疫抑制治疗容易并发感染，需要探索免疫抑制强度较轻的新疗法。

IMC患者自身免疫耐受丧失，自身抗体介导血细胞破坏增加或效应T细胞抑制骨髓造血细胞。在免疫抑制治疗失败后，探索免疫调节及促造血治疗可能获益。目前脐带血（umbilical cord blood, UCB）非移植输注治疗在血液病及非血液病中已经显示出其安全性和有效性[3]–[7]。脐带血中含有造血干祖细胞、间充质干细胞（MSC）以及一些未成熟的免疫细胞（调节性T细胞、NK细胞等），可能从不同角度对IMC有治疗作用。此外，人脐带血易于获得，可长期冷冻保存[8]。本研究采用异基因非亲缘非HLA匹配脐带血输注治疗12例难治性IMC患者，初步观察其安全性及疗效。

## 病例与方法

一、研究对象

本研究为前瞻性单臂临床试验（NCT04420494），纳入2020年9月至2021年10月期间中国医学科学院北京协和医院收治的IMC患者。入组标准：①年龄18～80岁；②诊断为AIHA（HGB<100 g/L）或PRCA（HGB<100 g/L）；③经过糖皮质激素治疗治疗4周未达到部分缓解（PR）及以上疗效；④无心功能异常、肝功能异常、肾功能异常，ECOG评分≤2分；⑤排除合并其他严重疾病和无法控制的全身感染的患者。本研究经中国医学科学院北京协和医院伦理审查委员会批准（ZS-2344），患者均签署书面知情同意书。

二、研究方法

1. 入组患者基线评估：采集患者基本信息、复核诊断，ECOG评分。实验室检查包括血常规、外周血涂片、网织红细胞、肝功能、肾功能、抗核抗体谱、乙肝病毒抗体及DNA、铁蛋白、维生素B12、叶酸、红细胞生成素（EPO）、Coombs试验、淋巴细胞亚群、免疫球蛋白定量、蛋白电泳、巨细胞病毒（CMV）DNA、EB病毒（EBV）DNA、细小病毒B19 DNA、骨髓细胞形态学、骨髓活检病理及流式细胞术免疫分型。

2. 合并用药：允许合并使用稳定剂量糖皮质激素，治疗后2个月内不允许增加剂量。脐带血输注前2周至输注后2个月不允许使用除了糖皮质激素之外的免疫抑制剂及免疫调节剂、静脉人免疫球蛋白（IVIg）。对于脐带血输注过程中出现呕吐、过敏等不良反应的患者，医生可根据具体情况使用止吐剂和抗过敏药物。

3. 脐带血输注方法：静脉输注ABO及Rh血型同型非亲缘脐带血，单个核细胞数（TNC）>1.0×10^7^/kg，间隔2周，共输注2次。非亲缘脐带血不要求HLA匹配度。脐带血中含有冻存剂（10％二甲基亚砜和10％右旋糖酐），输注前进行复温，经外周静脉输注，液量50 ml，5～10 min输毕。

4. 观察指标：①治疗前和治疗后2个月的症状、体征、血常规、血液生化指标、输血量等疗效相关指标。②输注相关反应、感染、出血发生率等安全性指标。

三、疗效判定标准

1. AIHA：完全缓解（CR）定义为HGB>120 g/L（男）或110 g/L（女），无溶血表现；PR定义为HGB升幅>20 g/L或HGB>100 g/L；病情稳定（SD）定义为HGB较脐带血输注前波动不超20 g/L。

2. PRCA：CR定义为HGB>120 g/L（男）或110 g/L（女），网织红细胞水平正常；PR定义为HGB升幅>20 g/L或HGB>100 g/L；SD定义为HGB较脐带血输注前波动不超过20 g/L。

四、随访及研究终点

患者随访资料来自门诊/住院病历和电话随访记录。随访截至时间为2022年8月。中位随访时间15（1～23）个月。主要研究终点为临床有效率，次要研究终点包括复发率、1年无病生存率、1年总生存（OS）率。

五、统计学处理

采用SPSS软件进行数据分析，双侧*P*<0.05被认为其差异显著有统计学意义。符合正态分布的定量指标用“均值±标准差”表示，非正态分布用“中位数（范围）”表示。分类指标使用“频数（百分比）”表示。患者治疗前后数据比较采取配对*t*检验，多组数据之间比较采用方差分析。计数资料的比较采用卡方检验。

## 结果

一、患者特征

12例患者中男6例，女6例，中位年龄53.5（30～78）岁，AIHA 8例（均为温抗体型），PRCA 4例。8例AIHA患者中5例Coombs试验阳性（未行Coombs分型鉴定），另3例为阴性。脐带血输注前中位病程为35（10～204）个月。入组前接受过糖皮质激素及多种免疫抑制剂治疗，中位治疗线数为3.5（1～6）线，患者临床特征及既往治疗用药见表1。

**表1:** 12例异基因脐带血输注治疗的免疫性血细胞减少症患者临床特征

例号	性别	年龄（岁）	诊断	脐带血治疗前病程（月）	既往治疗	合并用药	合并症	自身抗体
1	男	46	AIHA（Coombs试验阴性）	19	糖皮质激素，环磷酰胺，他克莫司，EPO	泼尼松20 mg/d（每周减2.5 mg，减量至15 mg/d维持）	无	阴性
2	男	52	AIHA（Coombs试验阴性）	204	糖皮质激素，西罗莫司，羟氯喹，EPO	无	类固醇糖尿病	ANA（+），β2GP1-IgM（+），ACL-IgM（+）
3	女	44	AIHA	30	糖皮质激素，环孢素A，利妥昔单抗	泼尼松15 mg/d×2周，7.5 mg/d×4周，5 mg/d×2周	右下肢肌间静脉血栓	ACL-IgM（+）
4	男	46	AIHA	34	糖皮质激素，硫唑嘌呤，环磷酰胺，利妥昔单抗	甲泼尼龙16 mg/d	2型糖尿病，肝功能异常	阴性
5	女	78	AIHA	10	糖皮质激素	泼尼松20 mg/d	2型糖尿病，哮喘，双侧股骨头坏死	ANA（+）
6	女	71	AIHA	21	糖皮质激素，环孢素A，西罗莫司，他克莫司	无	高尿酸血症	ANA（+）
7	男	72	AIHA	14	糖皮质激素，他克莫司	无	高血压，脑梗死	ANA（+），AMA-M2（+）
8	女	31	AIHA	54	糖皮质激素，利妥昔单抗，硼替佐米，硫唑嘌呤	泼尼松5 mg/d	类固醇糖尿病	阴性
9	男	75	PRCA	180	糖皮质激素，环孢素A，西罗莫司，IVIg，EPO，艾曲波帕，环磷酰胺，他克莫司	无	慢性肾功能不全，高血脂，高尿酸、痛风	阴性
10	男	30	PRCA	37	糖皮质激素，环孢素A，西罗莫司，IVIg	无	细小病毒B19 IgM（+），B19 DNA（−）	阴性
11	女	34	PRCA	43	糖皮质激素，环孢素A，西罗莫司，他克莫司，环磷酰胺，霉酚酸酯	甲泼尼龙8 mg/d	自身免疫性多内分泌腺病综合征	阴性
12	女	63	PRCA	60	糖皮质激素，环孢素A，利妥昔单抗，硼替佐米	无	大颗粒淋巴细胞白血病	阴性

**注** AIHA：自身免疫性溶血性贫血；PRCA：纯红细胞再生障碍；EPO：促红细胞生成素；IVIg：静脉注射免疫球蛋白；ANA：抗核抗体；AMA：抗线粒体抗体

二、脐带血输注

本组病例输注的脐带血均由山东省脐带血库提供。12例患者共完成22例次脐带血输注，其中2例患者因新冠疫情限制未能完成第二次输注。输注的脐带血中位单个核细胞（MNC）数为8.24（5.35～13.75）×10^8^，中位冻存血量32.6（23.1～48.2）ml，中位粒-巨噬细胞集落形成单位（CFU-GM）1.91（0.44～6.58）×10^6^，中位CD34^+^细胞数4.96（1.96～23.26）×10^6^，中位细胞活性84％（80％～92％），中位有核细胞计数14.89（11.41～17.66）×10^8^。脐带血采集和输注的中位间隔时间为24（13～44）个月。详见表2。

**表2:** 12例免疫性血细胞减少症患者输注的脐带血参数

例号	MNC［×10^8^（第1次，第2次）］	冻存血量［ml（第1次，第2次）］	CFU-GM［×10^6^（第1次，第2次）］	CD34^+^细胞［×10^6^（第1次，第2次）］	细胞活性［%（第1次，第2次）］	TNC［×10^8^（第1次，第2次）］	采集和应用间隔［月（第1次，第2次）］
1	8.21, 8.31	28.6, 34.7	2.15, 0.44	4.91, 2.78	92, 82	15.62, 14.23	15, 39
2	8.31, 8.07	38.5, 36.3	0.98, 2.02	4.12, 6.32	84, 80	14.51, 14.74	24, 24
3	8.46, 10.28	33.4, 29	1.66, 1.44	2.65, 5.52	83, 84	15.66, 15.83	24, 40
4	10.13, 6.46	34.3, 39	1.49, 2.77	3.03, 3.68	82, 83	12.55, 15.80	30, 32
5	10.01, 7.14	32.4, 32.9	2.31, 6.58	5.26, 9.55	85, 81	14.81, 15.96	40, 22
6	5.60, 7.17	31.2, 36.5	1.5, 1.22	2.32, 5.67	82, 82	12.67, 14.49	22, 27
7	5.62	28.6	3.35	9.27	85	12.44	24
8	5.67	26.9	3.79	5.01	83	11.41	19
9	11.54, 7.25	29.9, 29.6	3.71, 1.37	23.26, 1.96	85, 89	15.16, 14.98	24, 25
10	8.43, 8.28	35.9, 23.1	0.95, 6.49	3.95, 12.48	88, 84	14.14, 16.29	20, 13
11	7.17, 5.35	48.2, 31.3	1.07, 3.32	6.39, 3.47	88, 82	12.63, 15.12	44, 16
12	13.75, 9.49	29.3, 33.9	1.8, 2.64	8.86, 2.85	88, 84	16.79, 17.66	28, 18

**注** MNC：单个核细胞；CFU-GM：粒-巨噬细胞集落形成单位；TNC：总有核细胞数；例7、例8只完成1次脐带血输注

三、疗效评价

1例AIHA患者在脐带血输注后1个月失访，其余11例患者完成脐带血输注后2个月进行疗效评估。7例可评估疗效AIHA患者中6例为SD，1例为PR。4例PRCA患者中，SD、NR各2例。详见表3。

**表3:** 11例难治性免疫性血细胞减少症患者脐带血输注的疗效、挽救治疗及转归

例号	治疗前HGB（g/L）	治疗后HGB（g/L）	疗效	挽救治疗	随访时间（月）	转归
1	69	64	SD	西罗莫司，霉酚酸酯	13	不明原因死亡
2	76	97	PR	罗沙司他	22	HGB 90 g/L，疗效SD
3	99	87	SD	糖皮质激素，硫唑嘌呤，利妥昔单抗，硼替佐米	22	利妥昔单抗联合硼替佐米疗效CR，并发肺结核
5	91	86	SD	糖皮质激素，西罗莫司，利妥昔单抗	15	西罗莫司疗效SD，利妥昔单抗疗效CR
6	77	86	SD	EPO，Rilzabrutinib临床试验	13	HGB 90 g/L，疗效SD
7	80	72	SD	他克莫司	12	HGB 92 g/L，疗效SD
8	94	95	SD	西罗莫司	11	西罗莫司疗效CR
9	72	77	NR	IVIg，霉酚酸酯，罗沙司他	23	输血依赖
10	61	44	NR	西罗莫司，罗沙司他，IVIg	22	输血依赖
11	60	59	SD	糖皮质激素，霉酚酸酯，利妥昔单抗，硼替佐米	15	糖皮质激素维持HGB≥100 g/L，疗效SD
12	89	68	SD	西罗莫司，霉酚酸酯	22	西罗莫司治疗达CR

**注** EPO：促红细胞生成素；IVIg：静脉注射免疫球蛋白；NR：无效；SD：病情稳定；CR：完全缓解。随访时间：从第1次脐带血输注到末次随访的时间

四、挽救治疗及转归

所有患者在脐带血输注治疗后均进行了挽救治疗，4例患者挽救治疗后获得CR。3例AIHA患者分别经过利妥昔单抗联合硼替佐米、利妥昔单抗单药、西罗莫司单药治疗后获得CR，1例PRCA患者在西罗莫司治疗后获得CR。4例患者HGB稳定保持>90 g/L。2例患者持续输血依赖，均为PRCA患者（例9、例10）。1例AIHA患者（例1）因不明原因死亡，1例AIHA患者（例4）输注脐带血1个月后失访。挽救治疗及转归见表3。

五、不良反应

22例次脐带血输注均顺利完成。输注过程中心率、血压、血氧饱和度均稳定。1例患者输注过程中发生恶心（Ⅰ级），无呕吐，自行缓解。无过敏反应发生。随访期间未发生移植物抗宿主病（GVHD）。

## 讨论

本研究调查了难治复发AIHA和PRCA患者间隔2周静脉输注双份异基因非HLA匹配、无关供者脐带血的疗效和安全性。输注的脐带血中位MNC为8.24（5.35～13.75）×10^8^，中位CD34^+^细胞为4.96（1.96～23.26）×10^6^。达到治疗效果所需的剂量、输注方案尚不清楚，选择双份脐带血间隔2周输注耐受性良好，中位随访15（1～23）个月，未观察到GVHD发生。

AIHA和PRCA是IMC的主要类型。AIHA被认为是自身产生针对红细胞抗原的抗体导致红细胞过早破坏而生成相对不足，糖皮质激素是温抗体型AIHA的主要治疗手段[9]–[10]。PRCA的发病由细胞毒性T细胞破坏骨髓中红系前体细胞所致，环孢素A是最常用的免疫抑制剂[11]。两种疾病异质性很大，但共同的特征是免疫失调、免疫耐受丧失，免疫抑制治疗失败后，可尝试免疫调节和促造血治疗。

脐带血是造血干祖细胞的丰富来源，已被广泛用于异基因造血干细胞移植[3]–[12]。脐带血输注的目标不是干细胞植入。重型再生障碍性贫血患者强化免疫抑制治疗后行脐带血输注，可缩短反应时间、提高反应率[4]–[6]，患者并未发生供者干细胞植入，表明脐带血发挥了造血支持作用。脐带血输注还被尝试用于缺血性脑卒中[13]、自闭症谱系障碍[14]、创伤性脑损伤[15]–[16]等疾病的治疗。脐带血含有MSC、调节型T细胞、内皮前体细胞[17]以及一些未成熟的免疫细胞。MSC通过调节T细胞和B细胞的增殖和分化、树突状细胞成熟和自然杀伤活性来增强免疫抑制、发挥免疫调节功能[18]–[19]，已被用于GVHD[20]、类风湿性关节炎[21]等多种免疫相关疾病的预防和治疗。调节型T细胞主要发挥免疫抑制功能，已被尝试用于自身免疫性疾病的治疗[22]–[23]。基于以上研究成果，我们采用脐带血输注治疗免疫抑制治疗失败的AIHA和PRCA患者，期望其发挥免疫调节、免疫抑制的作用并提供干祖细胞支持。本研究22例次脐带血输注均未行HLA配型，所有患者均接受过糖皮质激素和多种免疫抑制剂治疗，均顺利完成输注，随访中未观察到感染和GVHD发生，未见其他明显不良反应，提示脐带血输注在复发难治AIHA和PRCA患者中具有良好的安全性。本研究中11例患者可评估治疗后2个月疗效，PR 1例、SD 6例、NR 4例，未获得CR疗效，有4例患者在经过挽救治疗后获得CR。以上提示单独脐带血输注可能不足以明显改变难治AIHA和PRCA的疾病状态，但限于开放标签和小样本的研究设计，上述结论尚需验证。

本研究结果显示，脐带血输注具有良好的安全性，但疗效并不理想。脐带血或脐带血来源免疫效应细胞输注联合免疫抑制治疗值得继续研究。
